# Spatiotemporal trends in COVID-19 vaccine sentiments on a social media platform and correlations with reported vaccine coverage

**DOI:** 10.2471/BLT.23.289682

**Published:** 2023-10-31

**Authors:** Xinyu Zhou, Xu Zhang, Heidi J Larson, Alexandre de Figueiredo, Mark Jit, Samah Fodeh, Sten H Vermund, Shujie Zang, Leesa Lin, Zhiyuan Hou

**Affiliations:** a School of Public Health, NHC Key Laboratory of Health Technology Assessment, and Global Health Institute, Fudan University, 130 Dong’an Road, Shanghai, 200032, China.; b Department of Infectious Disease Epidemiology, London School of Hygiene and Tropical Medicine, London, England.; c Department of Emergency Medicine, Yale School of Medicine, New Haven, United States of America (USA).; d Department of Epidemiology of Microbial Diseases, Yale School of Public Health, New Haven, USA.

## Abstract

**Objective:**

To assess spatiotemporal trends in, and determinants of, the acceptance of coronavirus disease 2019 (COVID-19) vaccination globally, as expressed on the social media platform X (formerly Twitter).

**Methods:**

We collected over 13 million posts on the platform regarding COVID-19 vaccination made between November 2020 and March 2022 in 90 languages. Multilingual deep learning XLM-RoBERTa models annotated all posts using an annotation framework after being fine-tuned on 8125 manually annotated, English-language posts. The annotation results were used to assess spatiotemporal trends in COVID-19 vaccine acceptance and confidence as expressed by platform users in 135 countries and territories. We identified associations between spatiotemporal trends in vaccine acceptance and country-level characteristics and public policies by using univariate and multivariate regression analysis.

**Findings:**

A greater proportion of platform users in the World Health Organization’s South-East Asia, Eastern Mediterranean and Western Pacific Regions expressed vaccine acceptance than users in the rest of the world. Countries in which a greater proportion of platform users expressed vaccine acceptance had higher COVID-19 vaccine coverage rates. Trust in government was also associated with greater vaccine acceptance. Internationally, vaccine acceptance and confidence declined among platform users as: (i) vaccination eligibility was extended to adolescents; (ii) vaccine supplies became sufficient; (iii) nonpharmaceutical interventions were relaxed; and (iv) global reports on adverse events following vaccination appeared.

**Conclusion:**

Social media listening could provide an effective and expeditious means of informing public health policies during pandemics, and could supplement existing public health surveillance approaches in addressing global health issues.

## Résumé

**Objectif:**

Évaluer les tendances spatiotemporelles et les facteurs d'acceptation de la vaccination contre la maladie à coronavirus 2019 (COVID-19) dans le monde, tels qu'exprimés sur le réseau social X (anciennement Twitter).

**Méthodes:**

Nous avons collecté sur la plateforme plus de 13 millions de publications consacrées à la vaccination contre la COVID-19, rédigées dans 90 langues entre novembre 2020 et mars 2022. Les modèles de deep learning multilingues XLM-RoBERTa ont annoté toutes les publications selon un cadre spécifique après avoir été réglés sur 8125 publications rédigées en anglais et annotées manuellement. Les résultats de ces annotations ont ensuite servi à évaluer les tendances spatiotemporelles en matière d'acceptation du vaccin contre la COVID-19 et le niveau de confiance affiché par les utilisateurs de la plateforme dans 135 pays et territoires. Enfin, nous avons identifié les liens entre ces tendances, les caractéristiques propres à chaque pays et les politiques publiques à l'aide d'une analyse de régression univariée et multivariée.

**Résultats:**

Dans les régions d'Asie du Sud-Est, de Méditerranée orientale et du Pacifique occidental de l'Organisation mondiale de la Santé, le pourcentage d'utilisateurs de la plateforme déclarant accepter le vaccin s’est révélé plus élevé que dans le reste du monde. La couverture vaccinale contre la COVID-19 était meilleure dans les pays où un plus grand nombre d'utilisateurs de la plateforme s’étaient montrés favorables à la vaccination. La confiance placée dans le gouvernement allait elle aussi de pair avec une meilleure acceptation du vaccin. À l'échelle internationale, l'acceptation et la confiance vis-à-vis du vaccin ont diminué chez les utilisateurs à partir du moment où: (i) les adolescents ont été considérés comme éligibles à la vaccination; (ii) l'approvisionnement en vaccins a été suffisant; (iii) les interventions non pharmaceutiques ont fait l'objet d'un assouplissement; et enfin, (iv) des rapports mondiaux sur les effets secondaires liés à la vaccination ont commencé à paraître.

**Conclusion:**

Le suivi des réseaux sociaux pourrait constituer un moyen rapide et efficace d'orienter les politiques de santé publique lors des pandémies. Il pourrait en outre s'ajouter aux dispositifs actuels de surveillance de la santé publique pour les questions de santé mondiales.

## Resumen

**Objetivo:**

Evaluar las tendencias espaciotemporales y los factores determinantes de la aceptación de la vacunación contra la enfermedad por coronavirus 2019 (COVID-19) a escala mundial, tal y como se expresan en la plataforma de redes sociales X (antes Twitter).

**Métodos:**

Se recopilaron más de 13 millones de publicaciones en la plataforma sobre la vacunación contra la COVID-19 entre noviembre de 2020 y marzo de 2022 en 90 idiomas. Los modelos multilingües de aprendizaje profundo XLM-RoBERTa anotaron todas las publicaciones mediante un marco de anotación después de haber sido ajustados en 8125 publicaciones en inglés registradas manualmente. Los resultados de la anotación se utilizaron para evaluar las tendencias espaciotemporales en la aceptación de la vacuna contra la COVID-19 y la confianza que expresaron los usuarios de la plataforma en 135 países y territorios. Se identificaron asociaciones entre las tendencias espaciotemporales en la aceptación de la vacuna y las características a nivel de cada país, así como las políticas públicas, mediante análisis de regresión univariante y multivariante.

**Resultados:**

Un mayor porcentaje de usuarios de la plataforma en las regiones de Asia Sudoriental, Mediterráneo Oriental y Pacífico Occidental de la Organización Mundial de la Salud expresaron su aceptación de la vacuna que los usuarios del resto del mundo. Los países donde un mayor porcentaje de usuarios de la plataforma expresaron su aceptación de la vacuna tuvieron mayores tasas de cobertura de la vacuna contra la COVID-19. La confianza en el gobierno también se asoció con una mayor aceptación de la vacuna. A nivel internacional, la aceptación de la vacuna y la confianza disminuyeron entre los usuarios de la plataforma a medida que: (i) la elegibilidad para la vacunación se amplió a los adolescentes; (ii) los suministros de vacunas fueron suficientes; (iii) se relajaron las intervenciones no farmacéuticas; y (iv) aparecieron informes globales sobre acontecimientos adversos tras la vacunación.

**Conclusión:**

El seguimiento de las redes sociales podría constituir un medio eficaz y rápido de orientar las políticas de salud pública durante las pandemias. También podría complementar los sistemas actuales de vigilancia de la salud pública para abordar los problemas sanitarios mundiales.

## ملخص

الغرض تقييم الاتجاهات الزمانية المكانية ومحددات قبول التطعيم ضد مرض فيروس كورونا 2019 (كوفيد 19) على مستوى العالم، كما هو مُعبر عنه على منصة التواصل الاجتماعي X (تويتر سابقًا).

الطريقة قمنا بجمع أكثر من 13 مليون منشور على المنصة بخصوص التطعيم ضد فيروس كورونا (كوفيد 19)، تم نشرها في الفترة ما بين نوفمبر/تشرين ثاني 2020، ومارس/آذار 2022 بـ 90 لغة. قامت نماذج XLM-RoBERTa للتعلم العميق متعدد اللغات بالتعليق التوضيحي على كل المنشورات باستخدام إطار التعليق التوضيحي بعد ضبطها بدقة على 8125 منشورًا باللغة الإنجليزية تم التعليق التوضيحي عليها يدويًا. تم استخدام نتائج التعليق التوضيحي لتقييم الاتجاهات الزمانية المكانية في قبول لقاح كوفيد 19 والثقة به، كما عبر عنها مستخدمو المنصة في 135 دولة ومنطقة. لقد حددنا الارتباطات بين الاتجاهات الزمانية المكانية في قبول اللقاح، والخصائص على مستوى الدولة والسياسات العامة باستخدام تحليل التحوف وحيد المتغير ومتعدد المتغيرات.

النتائج أعربت نسبة أكبر من مستخدمي المنصة في المناطق التابعة لمنظمة الصحة العالمية في جنوب شرق آسيا، وشرق البحر الأبيض المتوسط، وغرب المحيط الهادئ، عن قبولهم للقاح مقارنة بالمستخدمين في بقية أنحاء العالم. إن الدول التي عبرت نسبة أكبر فيها من مستخدمي المنصة عن قبول اللقاح، حققت معدلات تغطية أعلى للقاح كوفيد 19. وارتبطت الثقة في الحكومة أيضًا بقبول أكبر للقاحات. على المستوى الدولي، انخفض قبول اللقاح والثقة به بين مستخدمي المنصة نظرًا لما يلي: (1) تمديد أهلية التطعيم لتشمل المراهقين؛ و(2) أصبحت إمدادات اللقاح كافية؛ و(3) تم تخفيف التدخلات غير الصيدلانية؛ و(4) ظهور تقارير عالمية عن الآثار الجانبية التي أعقبت التطعيم.

الاستنتاج يمكن أن توفر الاستجابة لوسائل التواصل الاجتماعي وسيلة فعالة وسريعة لتوجيه سياسات الصحة العامة أثناء الأوبئة، ويمكن أن يدعم مناهج مراقبة الصحة العامة الحالية في معالجة قضايا الصحة العالمية.

## 摘要

**目的:**

评估社交媒体平台 X（前身为 Twitter）上表示的关于 2019 年冠状病（新型冠状病毒肺炎）疫苗接受程度在全球层面的时空趋势及其决定因素。

**方法:**

我们收集了平台上 2020 年 11 月至 2022 年 3 月期间用 90 种语言发布的有关新型冠状病毒肺炎疫苗接种的帖子，超过 1300 多万条。我们首先对 8125 篇英语帖子进行人工注解，使用人工注释数据集来训练和微调多语言深度学习 XLM – RoBERTa 模型，最后利用微调后的模型对所有帖子进行自动注解。注解结果用于评估 135 个国家和地区的平台用户表示的关于新型冠状病毒肺炎疫苗接受程度和信心的时空趋势。我们通过单变量和多变量回归分析确定了疫苗接受度的时空趋势与国家层面特征和公共政策之间的关联。

**结果:**

世界卫生组织东南亚、东地中海地区和西太平洋区域平台用户表示疫苗接受度的比例高于世界其他地区的用户。平台用户表示接受疫苗比例较高的国家，新型冠状病毒肺炎疫苗覆盖率也较高。疫苗接受程度较高也与对政府的信任有关。从全球层面看，平台用户对疫苗的接受程度和信心有所下降，原因如下：(I) 符合疫苗接种资格的群体扩大到青少年；(ii) 疫苗供应充足；(iii) 放宽了非药物干预措施的政策；以及 (iv) 出现了关于疫苗接种后不良事件的全球报告。

**结论:**

疫情期间，社交媒体监测可提供一种有效、快捷地传达公共卫生政策的方式，可作为解决全球卫生问题的现有公共卫生监测方法的补充。

## Резюме

**Цель:**

Оценить пространственно-временные тенденции и факторы, определяющие согласие на вакцинацию против коронавирусной болезни 2019 года (COVID-19) в глобальном масштабе, выраженные в социальных сетях на платформе X (ранее Twitter).

**Методы:**

В период с ноября 2020 г. по март 2022 г. на платформе было собрано более 13 млн публикаций о вакцинации COVID-19 на 90 языках. Многоязычные модели глубокого обучения XLM-RoBERTa аннотировали все публикации с использованием аннотационного фреймворка после доработки на 8125 вручную аннотированных англоязычных публикациях. Результаты аннотирования использовались для оценки пространственно-временных тенденций в приемлемости и доверии к вакцинации против COVID-19, выраженных пользователями платформы в 135 странах и территориях. С помощью одномерного и многомерного регрессионного анализа были определены ассоциации между пространственно-временными тенденциями в приемлемости вакцинации и характеристиками страны и государственной политикой.

**Результаты:**

Среди пользователей платформы в регионах Юго-Восточной Азии, Восточного Средиземноморья и Западного Тихоокеанского региона Всемирной организации здравоохранения большее число пользователей отметили приемлемость вакцинации по сравнению с пользователями в остальных странах мира. В странах, где большая доля пользователей платформы отметила приемлемость вакцинации, наблюдались более высокие показатели охвата вакцинацией против COVID-19. Доверие к правительству также было связано с большей приемлемостью вакцинации. В международном масштабе уровень приемлемости и доверия к вакцинации среди пользователей платформы снижался по мере того, как: (i) право на вакцинацию было распространено на подростков; (ii) запасы вакцины стали достаточными; (iii) нелекарственные вмешательства были смягчены; (iv) появились глобальные отчеты о нежелательных явлениях после вакцинации.

**Вывод:**

Прослушивание социальных сетей может стать эффективным и оперативным средством информирования о политике в области общественного здравоохранения во время пандемий и дополнить существующие подходы к эпиднадзору за состоянием здоровья населения при решении глобальных проблем здравоохранения.

## Introduction

Combatting a global pandemic, such as the coronavirus disease 2019 (COVID-19) pandemic, requires a multifaceted response from governments. Vaccination campaigns and nonpharmaceutical interventions, including city-wide lockdowns and travel restrictions, have a far-reaching impact on society and their effectiveness is contingent on public compliance. Consequently, policy-makers’ understanding of the impact of their decisions and the way they adjust policy in response to public concerns are key components of any effective public health intervention.

Monitoring data on social media through social media listening can play a crucial role in assisting policy-makers. By using advanced machine learning techniques, a nuanced narrative that reveals social attitudes, perceptions and actions can be constructed from simple textual (and visual) information on social media. Although social media users do not accurately represent the general population, geographical and temporal trends in their attitudes can reveal how the global or local social environment is reshaping people’s mindsets. In addition, social media listening enables researchers and policy-makers to scrutinize the ever-changing dynamics of the public’s response to public health measures in a cost-effective and expeditious way.^1^^,^^2^ Several previous studies have harnessed data from social media platforms, such as X (formerly Twitter; X Corp., San Francisco, United States of America) and Facebook (Meta Platforms, Cambridge, USA), to analyse acceptance of COVID-19 vaccines, principally in high-income countries such as Canada, the United Kingdom of Great Britain and Northern Ireland and the United States.^3^^–^^8^ In contrast, little is known about attitudes to vaccines in low- and middle-income countries.

In this study, we fine-tuned multilingual deep learning models to analyse posts on X (formerly tweets) on COVID-19 vaccination in 90 languages that were made between late 2020 and early 2022. We assessed global geographical and temporal trends in the acceptance of COVID-19 vaccines among platform users from 135 countries and territories, and validated our findings using statistical data on COVID-19 vaccination coverage. We also explored the determinants of trends in COVID-19 vaccine acceptance among platform users. The overall intention of our investigation of COVID-19 vaccine acceptance was to demonstrate how social media listening can be employed effectively in the public health domain.

## Methods

**Fig. 1:**
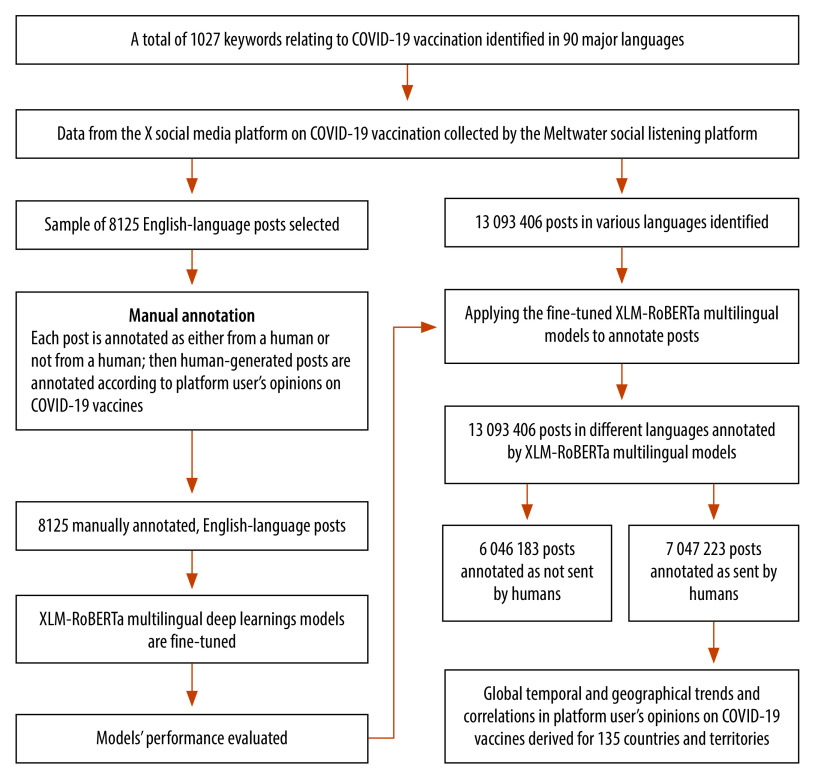
Flowchart, study of global trends in COVID-19 vaccine acceptance by social media platform users, November 2020 to March 2022

An overview of our data collection and analysis process is presented in Fig. 1 (full details of the methods are available from the online repository).^9^ To analyse posts on X multilingually, we developed an annotation framework for vaccine-related posts, which was first used by humans (i.e. not machines) to annotate a sample of posts. Then, we fine-tuned multilingual deep learning models to imitate human annotations and, finally, we annotated all posts available using the fine-tuned deep learning models.

COVID-19: coronavirus disease 2019.

### Collection of posts on social media platform X

We used the social media platform X as our data source because it is one of the world's most popular social media platforms. Between 2020 and 2023, the number of users worldwide fluctuated around 350 million. We identified 1027 keywords relating to COVID-19 vaccination that covered 90 major languages (details are available from the online repository).^9^ Using these keywords, we collected 13 093 406 publicly available posts on COVID-19 vaccination made in various languages between 13 November 2020 and 5 March 2022; these were all such posts identified by the Meltwater media monitoring and social listening platform (Meltwater, San Francisco, USA). We collected original and quote posts (i.e. secondary posts containing the original post with additional comments) but excluded simple secondary posts and replies.

### Annotation framework

We used the confidence, complacency and convenience (i.e. 3Cs) model of vaccine hesitancy proposed by the World Health Organization (WHO) to develop an annotation framework for COVID-19 vaccine-related posts,^10^ which was validated in a sample of 500 posts. Vaccine acceptance and vaccine refusal were the core measures. In addition, we investigated determinants of vaccine acceptance, such as confidence in vaccines, the online information environment and perceived barriers to accessing vaccines. Specifically, our annotation framework covered four key concepts related to COVID-19 vaccination and included eight categories (Table 1). First, COVID-19 vaccine acceptance covered the categories of: (i) intent to accept vaccination; and (ii) intent to refuse vaccination. Second, confidence in COVID-19 vaccines covered: (iii) belief that vaccines are effective; (iv) belief that vaccines are not safe; and (v) distrust in government. Third, the online information environment regarding COVID-19 vaccines covered: (vi) misinformation or rumours about vaccines. Fourth, perceived barriers to accessing COVID-19 vaccines covered: (vii) vaccine accessibility; and (viii) vaccine equity.

**Table 1:** Annotation categories for posts on vaccine hesitancy, study of global trends in COVID-19 vaccine acceptance by social media platform users, November 2020 to March 2022

Annotation category	Definition	Performance of deep learning models^a^	
		F_1_-score	Precision
**Vaccine acceptance**			
(i) Intent to accept COVID-19 vaccination	The post indicates that the platform user will accept, support or be willing to undergo COVID-19 vaccination	0.854	0.896
(ii) Intent to refuse COVID-19 vaccination	The post indicates that the platform user will refuse, will not support or will be unwilling to undergo COVID-19 vaccination	0.730	0.778
**Vaccine confidence**			
(iii) Belief that COVID-19 vaccines are effective	The post indicates that the platform user has confidence in the effectiveness of the COVID-19 vaccine	0.810	0.805
(iv) Belief that COVID-19 vaccines are not safe	The post indicates that the platform user lacks confidence in the safety of the COVID-19 vaccine	0.675	0.765
(v) Distrust in government	The post indicates that the platform user distrusts policy-makers or government bodies at any level (e.g. health ministries or centres for disease control)	0.792	0.727
**Online information environment**			
(vi) Misinformation or rumours about COVID-19 vaccines	The post contains negative information on vaccines, such as misinformation, rumours or references to anti-vaccine or anti-science campaigns or vaccine scandals	0.750	0.618
**Perceived barriers to accessing vaccines**			
(vii) COVID-19 vaccine accessibility	The post refers to production or supply limitations affecting the COVID-19 vaccine or the platform user’s ability to access vaccine	0.682	0.732
(viii) COVID-19 vaccine equity	The post refers to (priority) vaccination groups or to equity in vaccine allocation	0.809	0.859

COVID-19: coronavirus disease 2019.

^a^ The performance of deep learning models was assessed by comparing their annotations with annotations made by humans, with human annotation as the gold standard. The F_1_-score and precision are evaluation metrics widely used in machine learning (details are available in the online repository).^9^

Using this framework, two annotators independently annotated 8125 English-language posts on COVID-19 vaccination. Any disagreement was resolved by a third annotator. There were two main steps: (i) each annotator separated human-generated posts from news reports, advertisements, government announcements and posts generated by automated (i.e. bot) accounts; and (ii) each human-generated post was annotated according to its relevance to the eight annotation framework categories. A post could be relevant to one or more categories or to none. Examples of annotated posts are available in the online repository.^9^

### Fine-tuning multilingual deep learning models

Multilingual deep learning models are pretrained on textual data sets containing billions of words in multiple languages, and can develop a cross-lingual understanding of natural language.^11^ Fine-tuning these models using a small, manually annotated, task-specific data set in a single language enables them to perform the same task in around 100 languages without the need for translation.^12^^,^^13^

To analyse COVID-19 vaccine-related posts in 90 languages, we fine-tuned several multilingual deep learning models based on the recent, state-of-the-art, multilingual model, XLM-RoBERTa (HuggingFace, 2023),^14^ using our manually annotated, English-language data set. To do this, we randomly selected 80% of our 8125 manually annotated posts as a training set, 10% as a validation set and 10% as a retained test set (details are available from the online repository).^9^ The models learned how to annotate posts from the training set, and the validation set enabled us to determine hyperparameters (i.e. settings that influence how a machine learning model learns and performs). In the test set, the resulting deep learning models achieved a precision of 61.8% to 89.7% in automatically identifying human-generated posts and annotating them as relevant to the eight annotation framework categories (Table 1).

When applied to the 13 093 406 publicly available posts on COVID-19 vaccination in various languages, the fine-tuned XLM-RoBERTa models identified 6 046 183 posts as not sent by humans. The models then annotated the remaining 7 047 223 human-generated posts according to their relevance to the eight annotation framework categories.

### Statistical analysis

For each of the eight categories in our annotation framework, we derived the aggregate expressed opinion of each user on the platform by averaging all annotations of their posts made by the fine-tuned XLM-RoBERTa models within each specific time period. Then we evaluated the average aggregate expressed opinions of platform users over different time intervals and in different geographical locations. Time trends were evaluated using all human-generated posts. In contrast, geographical variations were evaluated using human-generated posts for which geolocation data were available from Meltwater, which uses a platform user’s profile data to derive the best estimate of their geographical location. We assessed geographical variations in 135 countries and territories with adequate data (details are available in the online repository).^9^

We used univariate and multivariate linear regression to identify determinants of COVID-19 vaccine acceptance and coverage across 135 countries and territories. In the regression analysis, we considered vaccination-related opinions on the platform X and 20 country variables, such as governance, pandemic preparedness, level of public trust, cultural factors (e.g. individualism), level of social development and demographic characteristics. Data on country variables were obtained from external sources (details are available from the online repository).^9^

Human-generated, vaccine-related posts were assessed on a daily basis, and spline regression was employed to fit global temporal trends in COVID-19 vaccine acceptance, vaccine confidence, the online information environment and perceived barriers to accessing vaccines. Country-level trends were assessed on a weekly and monthly basis for countries with sufficient data available. To explore determinants of temporal trends in vaccine acceptance, we constructed a country-level, weekly, panel data set that covered data on vaccine acceptance from posts on the platform and six indicators from external sources, including each country’s policies on vaccination and nonpharmaceutical interventions, and global reports of adverse events following immunization (details are available from the online repository).^9^ The panel data analysis employed a fixed-effects model.

All data analyses were performed using Python v. 3.7.2 (Python Software Foundation, Wilmington, USA) and R v.4.2.1 (The R Foundation, Vienna, Austria). The study was approved by the Institutional Review Board of the School of Public Health, Fudan University, Shanghai, China (IRB#2022–01–0938).

## Results

The XLM-RoBERTa deep learning models identified and annotated 7 047 223 human-generated posts on COVID-19 vaccination in various languages made between 13 November 2020 and 5 March 2022 by 3 344 144 platform users. Standardized, country-level, geolocation data were available for 4 137 550 (58.7%) of these posts, which were made by 1 953 157 platform users. Overall, 2 999 043 of the 3 344 144 X users (89.7%) made three or fewer posts on COVID-19 vaccination during the study period (details are available from the online repository).^9^ Of the 4 137 550 posts with geolocation data, 1 801 100 (43.5%) came from the United States; the United Kingdom accounted for 370 200 (8.9%); Canada for 250 001 (6.0%); Japan for 198 617 (4.8%); and India for 165 017 (4.0%). During the study period, the number of posts increased markedly in December 2020, when the first COVID-19 vaccine was approved, and decreased after January 2021 (details are available from the online repository).^9^

### Geographical variation

**Fig. 2:**
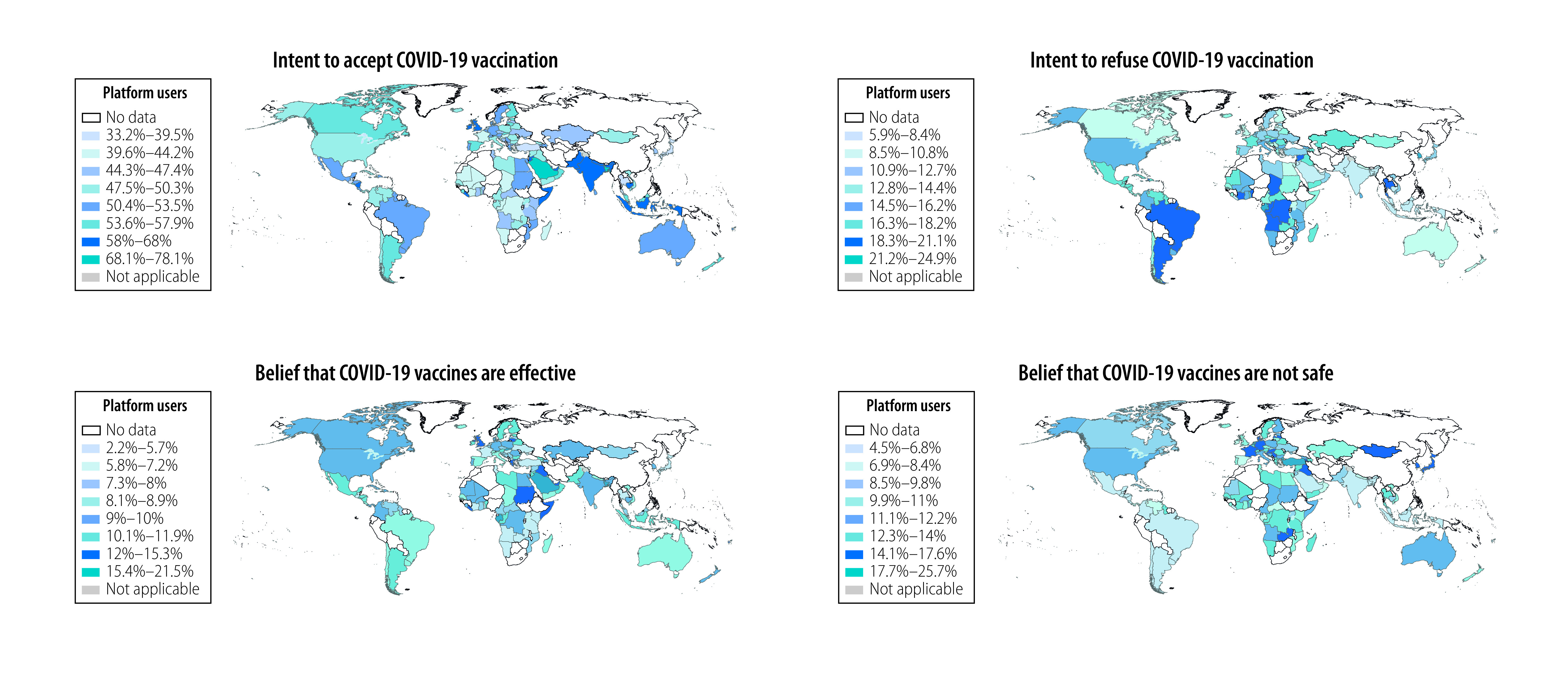
Geographical cumulative variation in COVID-19 vaccine acceptance, vaccine confidence, the online information environment and perceived barriers to accessing vaccines, as expressed by social media platform users, November 2020 to March 2022

Fig. 2 illustrates the proportion of social media platform users from different countries and territories whose posts were relevant to the eight annotation framework categories during the study period (full details are available from the online repository).^9^ Acceptance of, and confidence in, COVID-19 vaccines varied considerably across WHO regions: the proportion of platform users who expressed COVID-19 vaccine acceptance throughout the study period varied from 33.2% to 78.1% across countries and territories and the proportion who expressed an intention to refuse vaccination varied from 5.9% to 24.9%. Platform users in the South-East Asia, Eastern Mediterranean and Western Pacific Regions more often expressed vaccine acceptance and confidence in vaccine effectiveness and safety than users in the African Region, the Region of the Americas or the European Region. Countries in the South-East Asia Region accounted for four of the 10 countries or territories with the highest proportion of platform users who expressed vaccine acceptance: the proportion was 78.1% (772/989) of users in Bangladesh; 68.0% (62797/92 349) of users in India; 66.0% (1147/1738) of users in Nepal; and 64.3% (20 398/31 724) of users in Indonesia.

COVID-19: coronavirus disease 2019.

Notes: The maps show data for 135 countries and territories from each of which > 100 users posted on coronavirus disease 2019 vaccination between 13 November 2020 and 5 March 2022. The maps show the proportion of users who either expressed the opinion shown or commented on the topic shown. Countries or territories with inadequate data, whose governments blocked access to social media platform X or whose official languages were not fully included in the 90 languages analysed are depicted in white. Definition of each category is presented in Table 1.

Of the 10 countries or territories with the highest proportion of platform users who expressed an intention to refuse vaccination, four were from the Region of the Americas: the proportion was 24.4% (40/163) of users in Guadeloupe; 21.1% (25/120) of users in Martinique; 20.2% (123/611) of users in Haiti; and 19.6% (4369/22 295) of users in Argentina. An additional four were from the African Region: the proportion was 24.1% (25/104) of users in Réunion; 22.2% (23/102) of users in Gabon; 20.8% (98/474) of users in the Democratic Republic of the Congo; and 19.7% (30/153) of users in the Congo. Eight of the 10 countries with the highest proportion of users who expressed distrust in government were from the Region of the Americas: the proportion was 19.6% (479/2444) of users in El Salvador; 18.4% (1198/6514) of users in the Bolivarian Republic of Venezuela; 18.0% (15 665/87 031) of users in Brazil; 17.8% of (467/2624) users in Guatemala; 16.5% (2 577/15 618) of users in Colombia; 16.2% (202/1253) of users in Honduras; 16.0% (151/947) of users in Nicaragua; and 15.2% (4 673/30 745) of users in Mexico. Platform users in the African Region and the South-East Asia Region more often posted on vaccine accessibility and vaccine equity than users elsewhere.

### Determinants of country-level variation

Univariate linear regression found that, in aggregate, users’ opinions on vaccine confidence, the online information environment and perceived barriers to accessing COVID-19 vaccines were strongly associated with vaccine acceptance and refusal (full details are available from the online repository).^9^ The only country-level characteristics that had a significant positive association with vaccine acceptance were trust in government and internet coverage. Country-level characteristics that had a significant negative association with vaccine refusal included better governance, pandemic preparedness, trust in government and the level of social development. Multivariate regression, which controlled for other country-level characteristics, found that trust in government remained significantly associated with vaccine acceptance (Table 2 and Table 3). Furthermore, multivariate linear regression confirmed that users’ expression of vaccine acceptance was significantly associated with COVID-19 vaccination coverage at the country level (Table 4; full details are available from the online repository).^9^

**Table 2:** Country-level determinants of social media platform users expressing COVID-19 vaccine acceptance, on multivariate linear regression, study of global trends in COVID-19 vaccine acceptance by social media platform users, November 2020 to March 2022

Variable	Association with platform users’ intent to accept COVID-19 vaccination, regression coefficient (95% CI)
% of population with trust in government	0.149 (0.077 to 0.221)
% of population aged ≥ 65 years	−0.142 (−0.342 to 0.059)

CI: confidence interval; COVID-19: coronavirus disease 2019.

Notes: The multivariate linear regression analysis included variables for 78 countries or territories: (i) for which at least 65 data points were available; (ii) that were significant at the *P* < 0.1 level on univariate analysis; and (iii) that were free from multicollinearity (i.e. the correlation coefficient with another variable was < 0.8). If the correlation coefficient between two variables was ≥ 0.8, only one was included. Univariate linear regression results are available in the online repository.^9^

**Table 3:** Country-level determinants of social media platform users expressing COVID-19 vaccine refusal, on multivariate linear regression, study of global trends in COVID-19 vaccine acceptance by platform users, November 2020 to March 2022

Variable	Association with platform users’ intent to refuse COVID-19 vaccination, regression coefficient (95% CI)
**Pandemic preparedness**	
Global Health Security Index^15^	−0.014 (−0.117 to 0.089)
**Level of social development**	
% of school-aged children enrolled in school	0.015 (−0.029 to 0.059)
Sociodemographic index	−4.364 (−14.24 to 5.512)
**% of population with trust in government**	−0.045 (−0.094 to 0.004)
**Population density**	−0.002 (−0.007 to 0.004)

CI: confidence interval; COVID-19: coronavirus disease 2019.

Notes: The multivariate linear regression analysis included variables for 78 countries or territories: (i) for which at least 65 data points were available; (ii) that were significant at the *P* < 0.1 level on univariate analysis; and (iii) that were free from multicollinearity (i.e. the correlation coefficient with another variable was < 0.8). If the correlation coefficient between two variables was ≥ 0.8, only one was included. Univariate linear regression results are available in the online repository.^9^

**Table 4:** Country-level determinants of COVID-19 vaccination coverage, on multivariate linear regression, study of global trends in COVID-19 vaccine acceptance by social media platform users, November 2020 to March 2022

Variable	Association with COVID-19 vaccination coverage, regression coefficient (95% CI)
**Proportion of platform users expressing intent to accept COVID-19 vaccination**	0.791 (0.245 to 1.337)
**Fragility of nation state^a^**	−1.574 (−4.814 to 1.667)
**Pandemic preparedness**	
Global Health Security Index^15^	0.332 (−0.091 to 0.754)
Doctors per 1000 population	−0.130 (−0.498 to 0.239)
**Trust**	
% of population with trust in government	0.002 (−0.210 to 0.214)
% of population with trust in science	0.667 (0.215 to 1.118)
**% of school-aged children enrolled in school**	0.221 (0.038 to 0.403)
**Demographic characteristics**	
Population density	0.006 (−0.015 to 0.028)
% of population aged ≥ 65 years	−0.175 (−1.197 to 0.847)
% of population living in cities	0.264 (0.050 to 0.479)

CI: confidence interval; COVID-19: coronavirus disease 2019.

^a^ Fragility refers to the state’s incapacity to provide essential public goods and services and to cope with shocks.^16^

Notes: The multivariate linear regression analysis included variables for 78 countries or territories: (i) for which at least 65 data points were available; (ii) that were significant at the *P* < 0.1 level on univariate analysis; and (iii) that were free from multicollinearity (i.e. the correlation coefficient with another variable was ≥ 0.8). Univariate linear regression results are available in the online repository.^9^

### Temporal trends

**Fig. 3:**
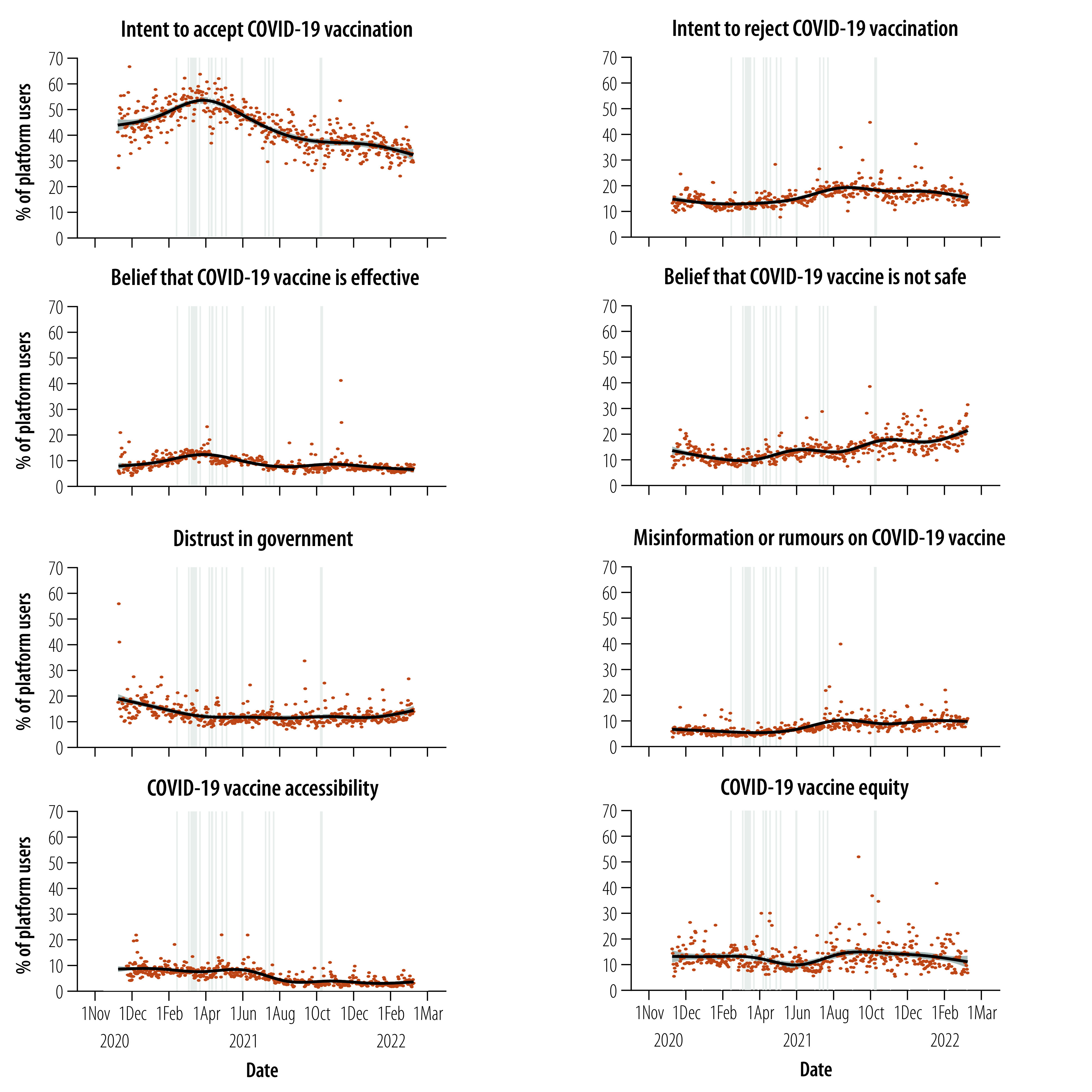
Global temporal trends in COVID-19 vaccine acceptance, vaccine confidence, the online information environment and perceived barriers to accessing vaccines, as expressed by social media platform users, November 2020 to March 2022

Fig. 3 shows daily temporal trends in COVID-19 vaccination-related opinions globally. Among platform users who commented on COVID-19 vaccination, the proportion who expressed acceptance of vaccination increased from a daily average of 44.1% in December 2020 to 56.0% in March 2021, and then declined slowly to reach a daily average of 33.4% in February 2022. The proportion who expressed refusal of vaccination climbed gradually from a daily average of 10.5% in February 2021 to 17.2% in August 2021, then remained stable. The proportion of users who expressed a belief in vaccine effectiveness peaked at 11.6% in March 2021 and then declined gradually to around 5.0% by February 2022. In parallel, the proportion who expressed a belief that vaccines were not safe rose gradually from a daily average of 9.6% in March 2021 to 18.9% in February 2022. The proportion who expressed distrust in government decreased throughout 2021. The proportion of users who posted about misinformation or rumours on COVID-19 vaccination generally increased during the observation period; in particular, there was a notable increase from a daily average of 4.3% in March 2021 to 10.4% in August 2021. Noteworthy is that vaccine acceptance and confidence began to decline as global reports of COVID-19 vaccine-related adverse events following immunization emerged after March 2021.

COVID-19: coronavirus disease 2019.

Notes: The graphs show daily global trends, as derived using spline regression, in the proportion of users who posted on coronavirus disease 2019 vaccination between 13 November 2020 and 5 March 2022. The days on which vaccine adverse events were reported are indicated by the vertical lines. Definition of each category is presented in Table 1.

**Fig. 4:**
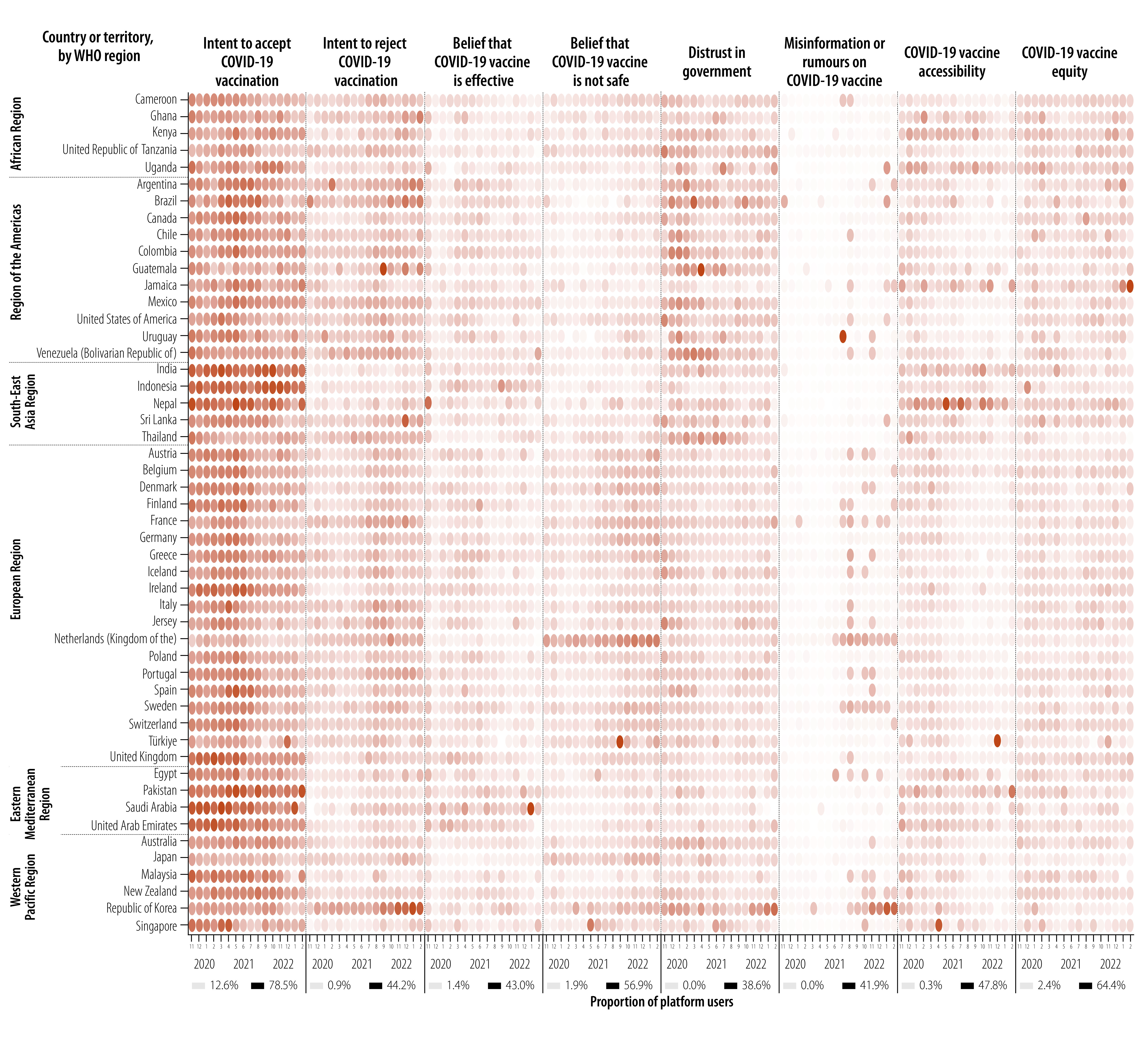
National monthly trends in COVID-19 vaccine acceptance, vaccine confidence, the online information environment and perceived barriers to accessing vaccines, as expressed by social media platform users, November 2020 to March 2022

COVID-19: coronavirus disease 2019; WHO: World Health Organization.

Notes: The illustration shows data for countries from which a total of ≥ 2500 users posted on coronavirus disease 2019 vaccination between 13 November 2020 and 5 March 2022, with posts from at least 30 users per month. However, Egypt and Nepal were included even though the total number of users posting in these countries was < 2500 but there were at least 30 users per month in each country. In total, data from 50 countries or territories are shown, with each point in the figure representing the monthly proportion of users who posted on each topic. Definition of each category is presented in Table 1.

The proportion of platform users who posted on COVID-19 vaccine accessibility remained largely stable until June 2021, at around a daily average of 8.5%, when it began to decline gradually. Posts on COVID-19 vaccine equity gradually increased in the second half of 2021 until the daily average proportion reached 17.4% in August 2021 and stabilized thereafter. Temporal trends at regional and country levels were similar to global trends (Fig. 4; additional details are available from the online repository).^9^

### Determinants of temporal trends

Table 5 presents the findings of a fixed-effects regression analysis that explored determinants of temporal trends in COVID-19 vaccine acceptance. The proportion of users whose posts expressed an intention to accept COVID-19 vaccination declined significantly when vaccination eligibility was extended to adolescents and vaccine supply became sufficient. In addition, the proportion of users who expressed an intention to refuse vaccination increased significantly when: (i) global reports of adverse events following immunization appeared; (ii) vaccination eligibility was extended to adolescents; and (iii) nonpharmaceutical interventions were relaxed.

**Table 5:** Determinants of temporal variation in social media platform users expressing COVID-19 vaccine acceptance, on panel data analysis, study of global trends in COVID-19 vaccine acceptance by social media platform users, November 2020 to March 2022

Variable	Regression coefficient (95% CI)	
	Association with temporal variation in platform users’ intent to accept COVID-19 vaccination^a^	Association with temporal variation in platform users’ intent to refuse COVID-19 vaccination^a^
Appearance of reports on adverse events following immunization	0.016 (−0.016 to 0.049)	0.014 (0.004 to 0.023)
Vaccine availability^b^	0.018 (−0.010 to 0.046)	−0.004 (−0.013 to 0.006)
Vaccination made mandatory	0.001 (−0.031 to 0.034)	−0.006 (−0.019 to 0.008)
Vaccination eligibility extended to adolescents	−0.052 (−0.088 to −0.016)	0.020 (0.008 to 0.033)
Vaccine supply became sufficient^b^	−0.080 (−0.113 to −0.047)	0.012 (−0.001 to 0.025)
Nonpharmaceutical interventions relaxed	−0.011 (−0.042 to 0.020)	0.018 (0.005 to 0.030)

CI: confidence interval; COVID-19: coronavirus disease 2019.

^a^ The panel data analysis, which involved 1716 country–week observations, employed a fixed-effects model and included lags on independent variables.

^b^ Vaccine availability implies that COVID-19 vaccine was available and the public was being vaccinated in the country during the week concerned. In contrast, the vaccine supply became sufficient when the COVID-19 vaccine supply was more than sufficient for the country's population.

Note: The analysis included data from only the 26 countries or territories with sufficient platform users’ posts on coronavirus disease 2019 (COVID-19) vaccination (i.e. total number of users > 5000, with a weekly number > 28 before the week of 20 February 2022 and > 18 thereafter).

## Discussion

Our study used multilingual social media listening to monitor geographical and temporal trends in opinions about COVID-19 vaccination expressed on the social media platform X. We assessed over 7 million human-generated posts from 135 countries and territories between the emergency approval of COVID-19 vaccines and the time when over half of the world’s population had been vaccinated. We found a promising association between the proportion of users who expressed COVID-19 vaccine acceptance and real-world vaccination coverage worldwide. We also found that vaccine acceptance was more common among users in WHO’s South-East Asia, Eastern Mediterranean and Western Pacific Regions than in the rest of the world, and that vaccine acceptance and confidence decreased as reports of adverse events following immunization emerged. These insights into geographical and temporal trends in vaccine acceptance could be valuable for devising proactive responses to potential vaccine hesitancy involving timely and targeted interventions.

Social media listening based on multilingual deep learning models can supplement the existing public health surveillance techniques used to address global health issues.^17^^,^^18^ This novel approach has several advantages: (i) monitoring can be conducted in real time, thereby enabling timely interventions; (ii) it is cost-effectivene and could be applied in low-resource settings, thereby improving research capacity and pandemic responses in low- and middle-income countries;^8^ and (iii) it could provide real-time insights into public sentiment to inform public health interventions, especially during outbreaks and pandemics.^8^ Unlike traditional research methods such as surveys, social media listening can rapidly and thoroughly scan the whole dynamic information environment for digital opinions derived from public contributions and interactions, without researcher involvement.^19^ Moreover, as it is not affected by the reporting bias that can result from interactions with researchers,^20^ social media listening can be particularly useful for research on sensitive public health issues.

Nevertheless, social media listening faces its own challenges, such as: (i) the potential non-representativeness of social media data; (ii) susceptibility to short-term noise (i.e. random fluctuations in opinion); (iii) a lack of demographic information; and (iv) a reliance on manually annotated data. First, there is an inherent bias in social media data because users may not express their genuine opinions online. Also, social media users are typically skewed towards younger individuals, who may be over-represented in anti-vaccine groups. Second, social media data are subject to short-term noise because emerging news may trigger disproportionate discussions on particular topics. Third, data on social media users’ demographic characteristics are generally unavailable, which limits in-depth analyses at the individual level. On the other hand, ecological analysis can be widely employed to identify associations at the population level, though it cannot infer causality. Fourth, social media listening based on deep learning models depends on domain-specific fine-tuning that relies heavily on the accurate incorporation of manual annotations.

Recognition of the merits and limitations of social media listening and its careful integration into public health surveillance are crucial for optimizing its effectiveness. Although platform users may not be representative of the general population, the young people and anti-vaccine groups concentrated on the platform still warrant attention from policy-makers. Social media listening can also be applied to other social media platforms, such as Facebook, Reddit and Instagram, which may be used by hard-to-reach population groups. In addition, social media’s sensitivity to news items and short-term events provides an opportunity to understand their impact on attitudes to vaccines. Moreover, social media analysis facilitates large-scale spatiotemporal analysis, which may not be possible with traditional surveillance approaches, such as surveys. Finally, our analytical approach can be adapted to incorporate multilingual versions of few-shot classification using pattern-exploiting training and SetFit (sentence transformer fine-tuning),^21^^,^^22^ which ensure good model performance even when manually annotated training data are scarce.

We found considerable geographical variation in COVID-19 vaccine acceptance as expressed on the platform, which was consistent with previous global surveys.^23^^–^^27^ We also found that vaccine acceptance on the platform could be a key predictor of vaccination coverage in the real world. In practice, the reliability of predictions based on social media listening could be verified by demonstrating consistency with surveys and real-world vaccination coverage. However, social media listening may produce underestimates of actual vaccination coverage, which has also been observed in survey-based studies.^28^^,^^29^ These underestimates may arise, in part, from compulsory vaccination policies: some vaccinated individuals with negative views about vaccination may express them on social media. In addition, the high prevalence of anti-vaccine groups on social media may skew online opinions about vaccine acceptance.

Our study highlighted the importance of trust in boosting vaccine acceptance and coverage, which is consistent with previous research suggesting that a high level of trust was associated with greater COVID-19 vaccine coverage and lower COVID-19 infection rates.^30^^,^^31^ Trust has also been associated with compliance with public health regulations, such as mask-wearing and observance of social distancing rules.^32^ Consequently, building trust in government is a priority for policy-makers seeking to promote compliance with public health interventions, including vaccination.

We observed a disturbing, continuous decline in COVID-19 vaccine acceptance and confidence after March 2021, when reports of adverse events following immunization emerged worldwide. This decline was also observed in previous surveys.^33^^,^^34^ The decline in vaccine acceptance presents a formidable challenge for policy-makers globally who depend on vaccination campaigns to combat pandemics and reduce preventable deaths.^33^^,^^35^ Policy-makers should proactively prepare to increase public support for vaccination in future pandemics, in addition to implementing public health surveillance.

Our study indicated that the main determinants of declining COVID-19 vaccine acceptance were: (i) the extension of vaccination eligibility to adolescents; (ii) a sufficient vaccine supply; (iii) the relaxation of nonpharmaceutical interventions; and (iv) reports of adverse events following immunization. Social media listening can provide an early indication of declining vaccine acceptance following changes in vaccination or nonpharmaceutical intervention policies, thereby enabling a prompt public policy response.

In summary, social media listening using machine learning can address complex public health issues across diverse settings and in many languages. We believe this is a new frontier for public health and medical surveillance that will provide policy-makers with near-real-time insights into public perceptions and views. Recognizing public fears and their origins is the first step in devising a rapid educational response. Insights from such surveillance can also help in anticipating similar fears in the future. In future pandemics, the acceptance of newly developed vaccines could be suboptimal and could decline, as occurred with COVID-19 vaccines. Consequently, key stakeholders and officials should make early preparations to ensure public support for vaccination.
